# Neurogenetic and Neuroepigenetic Mechanisms in Cognitive Health and Disease

**DOI:** 10.3389/fnmol.2020.589109

**Published:** 2020-12-03

**Authors:** Davide Martino Coda, Johannes Gräff

**Affiliations:** Laboratory of Neuroepigenetics, Brain Mind Institute, School of Life Sciences, École Polytechnique Fédérale de Lausanne (EPFL), Lausanne, Switzerland

**Keywords:** neuroepigenetics, neurogenetics, omics, gene regulatory networks, epigenetics, histone code

## Abstract

Over the last two decades, the explosion of experimental, computational, and high-throughput technologies has led to critical insights into how the brain functions in health and disease. It has become increasingly clear that the vast majority of brain activities result from the complex entanglement of genetic factors, epigenetic changes, and environmental stimuli, which, when altered, can lead to neurodegenerative and neuropsychiatric disorders. Nevertheless, a complete understanding of the molecular mechanisms underlying neuronal activities and higher-order cognitive processes continues to elude neuroscientists. Here, we provide a concise overview of how the interaction between the environment and genetic as well as epigenetic mechanisms shapes complex neuronal processes such as learning, memory, and synaptic plasticity. We then consider how this interaction contributes to the development of neurodegenerative and psychiatric disorders, and how it can be modeled to predict phenotypic variability and disease risk. Finally, we outline new frontiers in neurogenetic and neuroepigenetic research and highlight the challenges these fields will face in their quest to decipher the molecular mechanisms governing brain functioning.

**Graphical Abstract:**
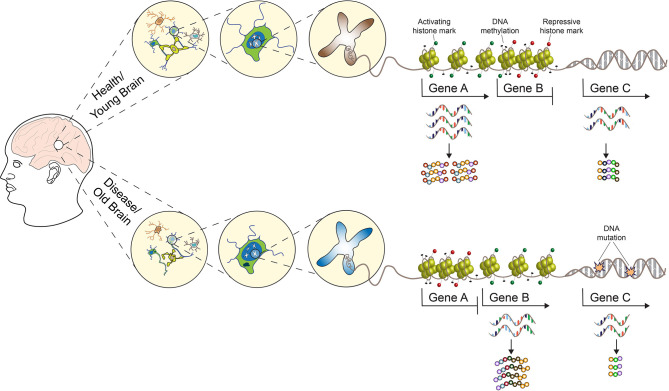


## Introduction: A Symbiotic Liaison Between Genes and the Environment

There are few areas of science more fiercely contested than the issue of what makes us who we are. How much of our identity is inherited, and how much acquired by interacting with the environment? Or, in other words, is nature the governing force shaping our personality, or is it nurture? While the eukaryotic genome is the same throughout all somatic cells in an organism, each expresses a unique set of genes that defines its specific identity. To describe the layer of mechanisms that resides above (*epi*) the level of the genes and that channels their outputs towards specific fates, the biologist Conrad Hal Waddington (1905–1975) conceived the term epigenetics in the early 1940s, defining it as “the study of the causal interactions between genes and their products which bring the phenotype into being” (Waddington, 1942). Since then, this field has continued to shed light on the entanglement of nature and nurture, genes and the environment, as during embryonic development, throughout the adult life, and in several diseases, cell-type-specific gene expression patterns are continuously established and maintained under the constant influx of intrinsic and extrinsic environmental cues by means of epigenetic modifications (Lee and Young, 2013; Cavalli and Heard, 2019).

In the last two decades, multiple lines of research have revealed that epigenetic mechanisms are also at play in the nervous system. These modifications stably alter gene activity in the context of the same genetic sequence, can self-sustain in the absence of the originating stimulus, and can be passed through cellular generations during neuronal lineage development (Gräff et al., 2011). At the same time, they are under environmental influence and can be modulated by internal and external stimuli, thus providing the cells with a system to rapidly encode and update information (Allis and Jenuwein, 2016). By having this Janus-faced property of being at once stable and malleable, epigenetic signatures emerged as an important mechanistic interface between life experiences and genome regulation in the brain (Jaenisch and Bird, 2003; Levenson and Sweatt, 2005; Hackman et al., 2010).

In addition to epigenetic mechanisms, we have been recently discovering the extent to which somatic mutations occurring during development and throughout the lifetime of an individual can affect human brain activities in physiological and pathological states. These non-inhered genetic changes are *de novo* mutations of the DNA sequence likely resulting from environmental insults such as inflammation and oxidative stress, as well as stochastic events (Nishioka et al., 2019). Ultimately, somatic mutations bring about a genetically heterogeneous population of neurons, whose identity is likely to be constantly shaped by the crosstalk of genetic and epigenetic mechanisms.

In this short review, we provide an overview of the multifaceted genetic and epigenetic regulation in the nervous system, discuss the state-of-the-art of neurogenetic and neuroepigenetic research and highlight its promises for a deeper understanding of brain functioning in health and disease.

## Established Principles: Genetic and Epigenetic Regulation of Gene Expression in The Brain

Genetic regulation occurs first at the level of the genetic code, which was deciphered shortly after the discovery of the DNA structure in 1953 (Watson and Crick, 1953). This code (Nirenberg and Leder, 1964; Marshall et al., 1967)—the set of rules by which information encoded in DNA sequences as nucleotide triplets are translated into proteins—came to its full appreciation upon the publication of the Human Genome Project in 2001, raising new hopes in the quest for understanding the basic principles across all physiological and pathological processes (Baltimore, 2001). Yet, despite this initial excitement, it soon became evident that the DNA alone was not able to generate the full range of information necessary to recapitulate the entire complexity of the human being and its diseases.

Around the same time, the histone code hypothesis gained widespread acceptance as a possible answer to this question. According to this hypothesis, the post-translational modifications of histone proteins, alone or in combination, would direct specific DNA-templated programs by (I) regulating the access of the transcriptional machinery to the underlying DNA sequences and (II) providing binding sites for effector proteins that selectively interact with distinct covalent histone marks (Jenuwein and Allis, 2001). For example, the acetylation of lysine residues on histone H3 and H4 by enzymes known as histone acetyltransferases (HATs) diminishes the electrostatic affinity between histone proteins and the DNA, promoting a chromatin structure that is more permissive to gene transcription, whereas the removal of the acetyl groups by enzymes known as histone deacetylases (HDACs) is associated with transcriptionally inactive chromatin (Kouzarides, 2007). Complicating matters further, chemical modifications of the DNA itself also play a role in the regulation of gene expression, with DNA methylation being the most studied example. Since such modifications of the DNA act in concert with histone modifications and do not occur independently of each other, it is paramount to extend the notion of a “histone code” to an “epigenetic code,” whereby specific patterns of epigenetic modifications regulate distinct gene expression networks within defined cell populations (Gräff and Mansuy, 2008).

In the field of neuroscience, the interaction between the genetic and epigenetic code has been best studied in the context of learning and memory. It was Francis Crick (1916–2004) in 1984 who first hypothesized that epigenetic modifications in terms of DNA methylation could store the memory of previously experienced stimuli, an idea that was later followed up by the molecular biologist Robin Holliday (1932–2014; Crick, 1984; Holliday, 1999). Accordingly, the repeated activation of neurotransmitter receptors during learning would trigger synapse-to-nucleus signaling which causes robust changes in gene expression, *via* epigenetic modifications. The downstream transcriptional responses result in the synthesis of new proteins, which in turn are used at the synaptic level to produce persistent changes in synaptic strength (Kandel, 2001). The first experimental evidence that epigenetic mechanisms could function as a signal-integration platform in the crosstalk between synapse and nucleus came from pioneering work in *Aplysia californica*. In this simple marine mollusk, synaptic plasticity—one of the neuronal mechanisms underpinning learning—requires the phosphorylation-mediated activation of the transcription factor (TF) cAMP-responsive element-binding protein 1 (CREB1). Once phosphorylated, CREB1 was found to recruit the HAT CREB-binding protein (CBP) to the promoter of the transcriptional co-activator CCAAT/enhancer-binding protein (CEBP), leading to enhanced histone acetylation and the expression of synaptic plasticity and memory-associated genes (Guan et al., 2002).

**Figure 1:**
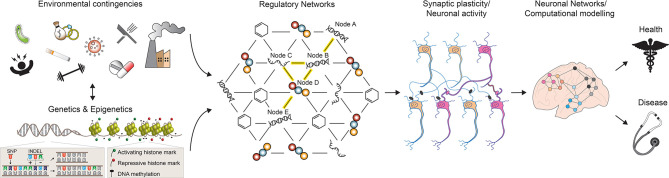
A multilevel model for understanding brain phenotypes. Brain states in both physiological and pathological conditions are the result of a complex array of interacting factors. Genetic, epigenetic, and environmental perturbations change the molecular states of regulatory networks controlling neuronal activities and functions. As a consequence, neuronal connections can be altered and existing synapses are strengthened or weakened. In turn, the structure and electrophysiological properties of the neuronal networks controlling higher-order brain functions are affected, leading to phenotypic changes towards health or disease. SNP, single nucleotide polymorphism; INDEL, insertion, and deletion. See the text for more details.

Since these first observations, many studies have confirmed the crucial involvement of histone acetylation, methylation, phosphorylation, and DNA methylation and their respective enzymes, as well as histone exchange processes to be implicated in learning, memory, and synaptic plasticity (Levenson et al., 2004; Miller and Sweatt, 2007; Koshibu et al., 2009; Gupta et al., 2010; Gräff and Tsai, 2013; Campbell and Wood, 2019), with new posttranslational histone modifications continuously being discovered (Farrelly et al., 2019; Zhang et al., 2019; Lepack et al., 2020). Besides learning and memory, a broad range of experiences ranging from psychological stress to nutrition and lifestyle were also found to induce epigenetic modifications in the central nervous system (CNS), and epigenetic mechanisms have been implicated in neurodevelopmental and neuropsychiatric disorders, neurodegeneration, and aging (see Figure 1 and the reviews, Krishnan and Nestler, 2008; Champagne and Curley, 2009; Gräff et al., 2011; Lyst and Bird, 2015; Hamilton and Nestler, 2019). Based on these findings, several drugs targeting the epigenome are currently in clinical trials with the hope to reverse genetically and/or environmentally induced aberrant epigenetic changes in the CNS. Examples of such epigenetically targeted drugs include the following: (a) HDAC inhibitors to treat Alzheimer’s disease (AD; NCT03056495 and NCT03533257), Parkinson’s disease (PD; NCT02046434), schizophrenia [NCT00194025, but note that the same drug—Valproate—has been previously tested for the treatment of AD (NCT00071721) with negative results] and cognitive decline (NCT02457507); (b) natural compounds which target DNA methyltransferase (Dnmt) activity to treat AD (NCT01716637 and NCT00951834). Despite these on-going efforts, the use of epidrugs for the treatments of neurodegenerative and neuropsychiatric disorders is still not suitable for routine clinical practice, and HDAC and DNMT inhibitors are currently FDA approved only for cancer therapy. The main factors that are preventing these drugs from achieving the same clinical success observed in the treatment of hematological malignancies and solid tumors can be ascribed to the CNS susceptibility to their genotoxicity, low stability, multi-targeted and multi-cellular effects, coupled with the fact that the epigenetic regulation of brain programs is highly heterogeneous and our understanding of the underlying principles currently limited (Szyf, 2015).

## State of The Art: Integrated “Omics” Approaches to Study Genetic and Epigenetic Variability and Disease Risk

Nowadays, genetic and epigenetic variability underlying a given phenotype is being studied by omics-based approaches. By sampling variations in the genome and epigenome in a large population of individuals and employing principles of statistical associations, risk factors, and likelihoods for neurodegenerative and neuropsychiatric disorders can be calculated (Diaz-Ortiz and Chen-Plotkin, 2020). The most widespread example of such approaches are GWAS, which aim at detecting associations between genetic variants, most often single-nucleotide polymorphisms (SNPs) across the entire genome and a phenotype of interest (Visscher et al., 2012). So far, large-scale GWAS have identified over 100 loci associated with frontotemporal lobar degeneration (FTLD), amyotrophic lateral sclerosis (ALS), AD, and PD for neurodegenerative diseases, as well as depression, addiction, schizophrenia, post-traumatic stress disorder (PTSD) and obsessive-compulsive disorders (OCD) for neuropsychiatric ones (Diaz-Ortiz and Chen-Plotkin, 2020). The major success to emerge from these studies was perhaps the identification of SNP markers linked to the *ApoE-ɛ4* allele as a risk factor for AD, hence replicating the association of the *ApoE-ɛ4* allele with AD originally proposed long before the advent of the genomic era (Corder et al., 1993; Coon et al., 2007; Bertram and Tanzi, 2009). ApoE is a major cholesterol carrier that supports lipid transport and injury repair in the brain, and different isoforms of ApoE have been shown to differentially regulate aggregation and clearance of amyloid β proteins (Aβ), crucial events for the development of AD (Kanekiyo et al., 2014). Despite the success of these studies in identifying genetic risk factors for neurodegenerative diseases and psychiatric disorders, these studies have not been without controversy. Prominent criticisms include the concerns that SNPs identified in GWAS explain only a small fraction of the heritability of complex traits, may represent spurious associations and have limited clinical predictive value (Tam et al., 2019). Furthermore, in the majority of cases it still remains unclear how the genetic variants identified by GWAS mechanistically and causally affect pathogenetic processes (Gandhi and Wood, 2010).

Indeed, the majority of such variants was found to occur in non-coding regions of the genome, making apparent how GWAS results need to be integrated with other layers of information to be correctly interpreted. Studies associating markers of genetic variation with gene expression data from hundreds of individuals have already identified loci at which genetic variation is statistically associated with the transcriptional levels of mRNAs of interest in disease-relevant tissues (Nica and Dermitzakis, 2013; Diaz-Ortiz and Chen-Plotkin, 2020). A notable example of so-called expression quantitative trait loci (eQTLs) comes from investigating the genetic variants linked to the *TMEM106B* gene in FTLD. Risk variants identified by GWAS associated with higher expression of *TMEM106B* through preferential recruitment of the chromatin-regulating protein CCCTC-binding factor (CTCF) and increased CTCF-mediated long-range interactions (Gallagher et al., 2017). In turn, increased *TMEM106B* expression resulted in lysosomal dysfunction in multiple cell types, including neurons, increasing the risk of developing cellular malfunctions and downstream neurodegeneration (Busch et al., 2016).

Another genome-wide means of identifying molecular events associated with human phenotypes recently emerged with EWAS. So far, EWAS have shown that complex diseases, in addition to genetic predispositions, also result from non-genetic risk factors likely mediated by epigenetic mechanisms (Birney et al., 2016). Indeed, a distinct pattern of DNA methylation at CpG dinucleotides enables us to discriminate between affected cases and control individuals in different pathological contexts (Rakyan et al., 2011). Such differentially methylated CpG sites that are dependent on genetic variants have been termed methylation quantitative trait loci (mQTLs), and have now been reported to occur in several neurological and neuropsychiatric disorders (Lord and Cruchaga, 2014; Roubroeks et al., 2017). Moreover, the integration of GWAS and EWAS with transcriptional profiling has revealed how loci harboring genetic variants can influence the methylation state of other loci *in cis* or *in trans*, which in turn correlates with different levels of gene expression (Do et al., 2017). For instance, in patients with AD, SNPs at an enhancer of the gene peptidase M20 domain-containing protein 1 (PM20D1) significantly correlated with levels of *PM20D1* DNA methylation and gene expression through a CTCF-mediated chromatin conformation change. People at risk for AD showed higher PM20D1 promoter methylation and reduced expression, while SNP carriers with reduced risk for AD displayed higher levels of PM20D1, which bestowed neuroprotection (Sanchez-Mut et al., 2018).

Overall, if we consider the entire collection of such large-scale omics-type studies performed so far, it is becoming clear that most common diseases are not the consequence of single genetic changes with a single outcome, but rather the result of perturbations of GRNs which are affected by complex genetic and environmental interactions (Figure 1). In their simplest representation, GRNs can be visualized as graphical models with two components: the nodes, which depict the molecular entities (DNA, transcripts, proteins, metabolites) observed to vary in the population under study, and the edges between nodes, which represent the physical and regulatory relationships between the molecular entities (Schadt, 2009). For example, a DNA node in a network can represent a SNP that modulates the expression of a gene in its proximity on the genome (node A and B in Figure 1); in turn, the resulting gene products (RNA and protein) control the activity of a second, long-distant gene (node C, D, and E in Figure 1). Essentially, as a consequence of multiple feedforward and feedback interactions between different biological substrates, the initial* cis*-regulatory event—a SNP affecting the expression of a neighboring gene—can also transmit its signal in *trans*, leading to changes in the activity of a gene located further apart on the genome (Nica and Dermitzakis, 2013). Once the components of a GRN have been identified, different approaches can be employed to model GRN dynamics and to predict its response to various environmental changes, both external and internal (Schlitt and Brazma, 2007). Under these premises, it is possible to imagine a nearby future in which the computation of such comprehensive networks of interacting molecular entities will greatly enhance our understanding of phenotypic variability and disease risk.

## Current Research Gaps and Future Directions

As a result of such research efforts, an extraordinary wealth of data is nowadays available to neuroscientists in the fields of neurogenetics and neuroepigenetics. Nevertheless, a unified framework in which these multi-modal and multi-scale data can be related, interpreted, and explored is still missing. To achieve a comprehensive and exhaustive understanding of brain functioning, we identify three major challenges that need to be surmounted: (1) refined measurement; (2) functional validation; (3) integrated computational modeling.

We refer to refined measurement as the need to complement fundamental observations coming from large populations of individuals or multiple brain regions with tissue-specific and cell-type-specific analyses. A promising approach to achieve this resolution is the use of single-cell sequencing-based technologies, which enable us to capture multiple features of individual cells with high-throughput methods (for details, see Table 1). Indeed, single-cell whole-genome amplification (scWGA) and single-cell RNA-sequencing (scRNA-seq) have already provided precious insights into the genomic and transcriptional variability of brain cells in different physiological and pathological contexts (Lodato et al., 2015; Ofengeim et al., 2017). At the same time, it is reasonable to expect that recently developed techniques allowing to profile the epigenomic state of single cells will also become of widespread use in neuroscience. Amongst these, the most informative approaches appear to be single-cell assay for transposase accessible chromatin (scATAC-seq), single-cell chromatin immunoprecipitation followed by sequencing (scChIP-seq) and single-cell bisulfite sequencing (scBS-seq), which measure—respectively—variations in chromatin accessibility, histone mark dynamics and DNA methylation in individual cells (Smallwood et al., 2014; Buenrostro et al., 2015; Grosselin et al., 2019). Also, proteomics and metabolomics tools able to assay different types of functional proteins and metabolites in individual cells have now started to be developed (Su et al., 2017). As a result of all these technological advances, it should become possible to leverage integrated analyses based on high-throughput sequencing to simultaneously capture the genomic, transcriptomic, epigenomic, and proteomic complexity of single cells. Generating this type of data will pave the way towards a more precise understanding of the molecular mechanisms underlying neuronal functions in both physiological and pathological contexts. For example, we now know that distinct changes in DNA methylation can function as an “epigenetic clock” to predict the biological age of an organism, and large DNA methylation datasets have already enabled accurate age estimates from different tissue across the life cycle including the brain (Horvath and Raj, 2018). With other types of epigenetic modifications as biomarkers of aging under investigation and their combined use with other omics-based approaches, we anticipate that it will become increasingly possible to identify molecular targets for interventions capable of slowing, halting, or even reversing brain aging processes, and even neuropsychiatric diseases.

**Table 1:** Summary of major genetic, epigenetic and transcriptional profiling techniques.

Technique	Type of assay	Single-cell profiling	Used in population studies	High-throughput	References
Whole-genome amplification	Detect single nucleotide polymorphisms (SNP) and copy number variants (CNV) across the genome	Yes	Yes	Yes	Lodato et al. (2015)
Chromatin immunoprecipitation using sequencing (ChIP-seq)	Capture protein-DNA binding events and posttranslational histone modifications genome-widely	Yes	No	Yes	Grosselin et al. (2019)
Assay for transposase accessible chromatin using sequencing (ATAC-seq)	Determine Chromatin accessibility across the genome	Yes	No	Yes	Buenrostro et al. (2015)
Whole-genome bisulfite sequencing (WGBS)	Determine the DNA methylation status of single cytosines across the genome	Yes	Yes	Yes	Karemaker and Vermeulen (2018)
Compartment analysis of temporal activity by fluorescence *in situ* hybridization (catFISH)	Visualize the subcellular localization of the mRNA of interest to infer neuronal activity	Yes	No	No	Guzowski et al. (2001)
Global run-on and sequencing (GRO-seq)	Measure nascent RNA production	No	No	Yes	Stark et al. (2019)
RNA sequencing (RNA- seq)	Measure steady-state mRNA levels	Yes	Yes	Yes	Stark et al. (2019)
Hi-C	Map chromatin contacts and interactions genome-wide	Yes	No	Yes	Hakim and Misteli (2012)
Chromatin interaction analysis by paired-end tag sequencing (ChIA-PET)	Identify genome-wide long-range chromatin interactions bound by protein factors	Yes	No	No	Hakim and Misteli (2012)

With the exceptional level of detail in the measurement of genetic and epigenetic changes on the horizon, it begs the question to what extent these variations causally contribute to the phenotypes under study. Such functional validation of (epi)genetic variation has recently moved within experimental reach thanks to a technique that allows to edit—directly *in vivo* in the brain as well as *in vitro* in cellular models—the genetic or epigenetic material at specific sites of interest in the genome, namely clustered regularly interspaced palindromic repeat (CRISPR)-Cas9 (Heidenreich and Zhang, 2016). With this technology, by using short fragments of RNA as “guides” for the Cas9 enzymatic machinery, nucleotide sequences at desired target sites in the genome can be removed, added, or altered with hitherto unachieved precision (Doudna and Charpentier, 2014). Excitingly, by using deactivated versions of the Cas9 enzyme (dCas9), epigenetic modifications at specific chromatin loci can also be altered. For example, fusing dCas9 to HAT or HDAC proteins like p300, CBP or HDAC3 enables site-specific control of histone acetylation levels; similarly, versions of dCas9 fused to the enzymes Tet1 and Dnmt3a allow for targeted DNA methylation editing events (Brocken et al., 2018; Xu and Qi, 2019). As their efficiency and scalability increases, we envision CRISPR-Cas9-based methodologies to become essential tools to validate the relevance of the genetic and epigenetic variances identified in GWAS and EWAS studies. At the same time, dCas9 systems—used individually or in combination—could be employed to shed light on the importance of the histone and epigenetic code for cognition and behavior in both health and disease. Indeed, while it is well established that distinct patterns of epigenetic modifications regulate specific gene expression networks during learning and memory, the underlying mechanisms remain to be elucidated (Campbell and Wood, 2019). Furthermore, as novel histone modifications—such as histone serotonylation, lactylation, and dopaminylation—keep being discovered, we anticipate that dCas9 versions fused to the enzymes mediating these modifications could be highly informative to characterize their roles in different brain functions (Farrelly et al., 2019; Zhang et al., 2019; Lepack et al., 2020).

Last, advances in the field of computational neuroscience are already allowing to build incredibly detailed models of neuronal connections and functioning within and across different brain areas, and such programs are currently already underway on different continents. By gathering information on the anatomical structure, electrophysiological properties, spatial positions, and connections of neurons, several large-scale brain simulators have been developed and keep being refined. So far, these brain models can be employed to successfully mimic synaptically connected networks of hundreds, thousands, or even more neurons, but are still unable to predict sensory stimulations or human behaviors (Einevoll et al., 2019). At the same time, these projects have predominantly focused on linking the neuronal to the network level, but the contribution of epigenetic/transcriptional dynamics to synaptic plasticity and neuronal function has yet to be taken into account. As a consequence, at present, a universal framework for brain activities able to directly connect—and thereby model—cognitive functions over synaptic networks down to genetic and epigenetic mechanisms are still missing (Einevoll et al., 2019). In this regard, it is worth noting that the concept of GRN has recently been expanded to encompass not only relationships among genes, epigenetic modifications, transcripts, proteins, and metabolites, but also intra- and intercellular communication, electrophysiological properties, and higher-order phenotypes such as learning and memory, and as such might provide a fundament for further integrating computational models (Baran et al., 2017). We are convinced that including nuclear dynamics in future modeling efforts would allow for a major step forward towards a better understanding of neuronal functioning.

Taken together, the future of neurogenetic and neuroepigenetics research lies, in our opinion, both in ever more refined (i.e., cell-type-specific) levels of analysis and in integrative, holistic approaches reaching beyond the nucleus. Within an individual, the combination thereof is poised to lead to apprehension for the full complexity of the multi-leveled structure of brain functioning, or malfunctioning. Within a population, such a research approach will be able to single out individuals at risk or bay for neurodegenerative and neuropsychiatric conditions and thereby pave the way for more personalized treatment approaches. Most importantly, this research endeavor will not only foster our understanding of how our brain functions but ultimately also of who we are.

## Author Contributions

DMC and JG wrote the article. All authors contributed to the article and approved the submitted version.

## Conflict of Interest

The authors declare that the research was conducted in the absence of any commercial or financial relationships that could be construed as a potential conflict of interest.

## Glossary

Epigenetics: originally defined by Conrad Waddington as “the study of the processes by which the genotype brings the phenotype into being,” it is now circumscribed as “the study of molecules and mechanisms that can perpetuate alternative gene activity states in the context of the same DNA sequence.”

Gene Regulatory Networks (GRNs): models used to describe and predict dependencies between molecular entities. A GRN identifies the set of genes that interact with each other through their RNAs and protein expression products to control a specific cell function.

Genome-wide association studies (GWAS)/Epigenome-wide association studies (EWAS): genome-wide approaches analyzing the statistical association between a molecular trait or disease and genetic variants (GWAS) or epigenetic marks (EWAS). The majority of GWAS examine single nucleotide polymorphisms (SNPs) across a large population of individuals, whilst EWAS measure their DNA methylation levels.

Histone code: the hypothesis according to which post-translational modifications of histones, alone or in combination, function to direct DNA-templated programs of gene expression by regulating binding site accessibility for specific effector proteins.

Omics: the comprehensive study of a group of distinct molecules using high-throughput technologies. Omics-based approaches are aimed at the detection of genes (genomics), epigenetic modifications (epigenomics), transcripts (transcriptomics), proteins (proteomics), and metabolites (metabolomics) in a specific biological sample in a non-targeted and non-biased manner.
